# Efficacy of immune checkpoint inhibitor combination therapy prior to nephrectomy in advanced renal cell carcinoma: A retrospective pilot study

**DOI:** 10.1002/bco2.419

**Published:** 2024-08-15

**Authors:** Sho Kiyota, Takashi Yoshida, Takahiro Nakamoto, Eri Jino, Takao Mishima, Hidefumi Kinoshita

**Affiliations:** ^1^ Department of Urology and Andrology Kansai Medical University Osaka Japan; ^2^ Department of Urology Osaka Saiseikai‐Noe Hospital Osaka Japan; ^3^ Graduate School of Engineering Tottori University Tottori Japan; ^4^ Corporate Sponsored Research Programs for Multicellular Interactions in Cancer Kansai Medical University Osaka Japan

**Keywords:** cytoreductive surgery, immune checkpoint inhibitor, metastatic tumour, progression‐free survival, renal cell carcinoma, unresectable tumours

## Abstract

Renal cell carcinoma (RCC) affects 10%–20% of patients annually, often with metastases present. This study evaluated the impact of systemic therapy before nephrectomy in patients with unresectable or metastatic renal cell carcinoma (RCC). Patients receiving upfront immune checkpoint inhibitor (ICI) combination therapy showed significantly improved progression‐free survival (PFS) compared to nephrectomy alone (2‐year PFS: 62.3% vs. 17.4%; *p* = 0.036), while upfront tyrosine kinase inhibitor (TKI) therapy did not (2‐year PFS: 18.2% vs. 12.3%; *p* = 0.545). Surgery‐related outcomes did not differ significantly between groups. ICI therapy maintained tumour reduction rates better than TKI therapy. The study highlights the potential benefits of ICI combination therapy over TKI therapy in advanced RCC, suggesting further research is needed to confirm these findings.

Approximately 10%–20% of patients diagnosed annually with renal cell carcinoma (RCC) present with metastatic disease while remaining the primary tumour.^1^ Neoadjuvant systemic therapy can lead to tumour size reductions, pathological necrosis during radical or cytoreductive surgery, and prognostic improvement in patients with unresectable or metastatic RCC. However, previous studies mostly assessing the impact of VEGFR‐targeted (TKI) therapy have yielded conflicting results,^2^, ^3^ and there is still a lack of such data in the era of immune checkpoint inhibitor (ICI) combination therapy.^4^, ^5^ Therefore, we sought to evaluate the impact of the transition from TKI therapy to ICI combination therapy on perioperative events and prognosis in patients with unresectable or metastatic RCC. This pilot study of advanced RCC, aimed to determine whether neoadjuvant systemic therapy, comprising either upfront TKI therapy (uTKI) or upfront ICI combination therapy (uICI), followed by nephrectomy, resulted in improved surgical and oncological‐related outcomes when compared to upfront nephrectomy (uN).

We conducted a retrospective review of 96 patients diagnosed with unresectable or metastatic RCC who underwent nephrectomy from January 2006 to December 2022. Unresectable RCC was defined as cases where the cancer was suspected not to achieve complete resection,^6^ such as disease with ≥cT3a with tumour thrombus or more and without metastasis. The histological subtypes included clear cell RCC (*n* = 77), unclassified RCC (*n* = 17), papillary RCC (*n* = 1), and mucinous tubular and spindle cell carcinoma (*n* = 1). Patients were divided into three groups: those treated with uN (*n* = 73) with or without subsequent systemic therapy, uTKI followed by nephrectomy (*n* = 12), and uICI followed by nephrectomy (*n* = 11). The clinicopathological variables of the study cohort are presented in Figure 1A.

**FIGURE 1:**
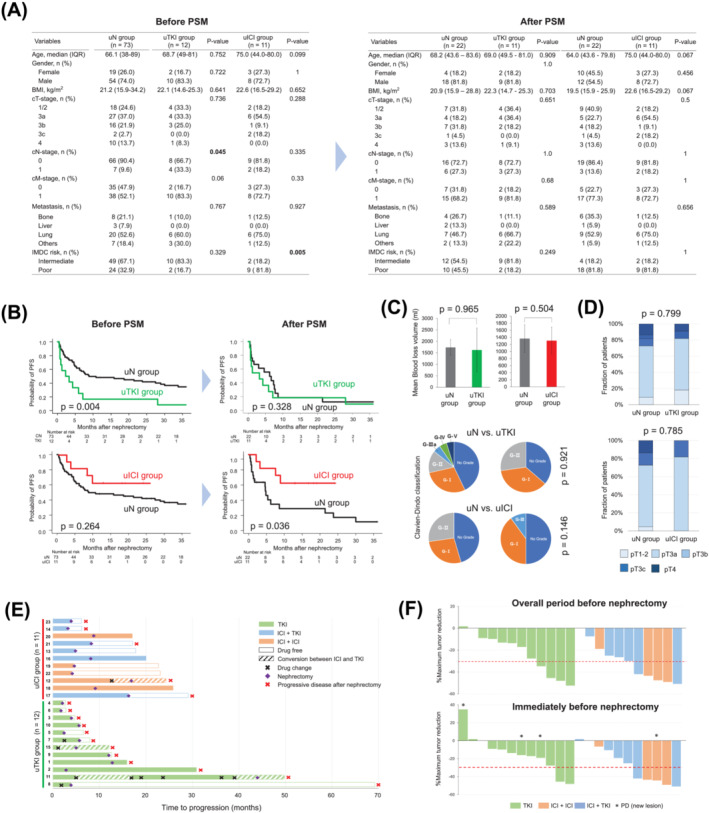
(A) Comparison of patient characteristics between the upfront nephrectomy (uN) group versus upfront VEGF‐targeted therapy (TKI) group or upfront immune checkpoint inhibitor combination therapy (uICI) group. A table on the left indicates variables before propensity score matching (PSM), and a table on the right indicates variables after PSM. Chi‐square or Mann–Whitney *U* test for statistical analysis. BMI, body mass index; IMDC, International Metastatic RCC Database Consortium; IQR, interquartile range. (B) Kaplan–Meier curves depict progression‐free survival, comparing the uN group versus the uTKI group and the uN group versus the uICI group. Panels on the left show data before propensity score matching (PSM), and those on the right show data after PSM. Log‐rank test was used for analysis. (C,D) Comparison of surgical‐related factors, including blood loss volume (mean value ± standard error) during nephrectomy and postoperative complication grade based on the Clavien–Dindo classification, and surgical pathology obtained by nephrectomy was performed between the groups after PSM. Chi‐square or Mann–Whitney *U* test for statistical analysis. (E) Swimmer plot of clinical course of all patients treated with either VEGF‐targeted therapy or immune checkpoint inhibitor combination therapy (*n* = 23; before PSM). (F) Best response and final response before nephrectomy for target lesions based on the maximal percentage of tumour reduction. PD, progression disease.

When determining treatment selection for unresectable or metastatic RCC at our institution, the decision between uN and uTKI was made at the physician's discretion until the introduction of combinational ICI therapy in Japan in August 2018. Since then, uICI has become the primary treatment modality for such eligible patients. For metastatic cases, deferred cytoreductive nephrectomy was generally performed in patients with good performance status and a limited metastatic burden.^7^

To minimize bias in patient characteristics between those who received each systemic therapy and those who did not (cN‐stage for uN vs. uTKI and the International Metastatic Renal Cell Carcinoma Database Consortium criteria for uN vs. uICI), we employed propensity score matching with a 1:2 matching protocol (greedy‐matching algorithm). The progression‐free survival (PFS) duration after the surgery was defined as de novo distant metastasis for M0/M1 cases or >20% enlargement of metastatic lesions for M1 cases. Survival analysis was performed using the Kaplan–Meier method with the log‐rank test. The study was approved by the Ethics Committee of Kansai Medical University (approved number: 2018109).

The types of agents used in the study were as follows: TKI agents included sorafenib (*n* = 2), sunitinib (*n* = 6), pazopanib (*n* = 3), and cabozantinib (*n* = 1); ICI combination agents comprised nivolumab plus ipilimumab (*n* = 5), pembrolizumab plus axitinib (*n* = 4), and avelumab plus axitinib (*n* = 2). After propensity score matching, 22 patients in the uN group and 11 patients in the uTKI group were included, as well as 22 patients in the uN group and 11 patients in the uTKI group, with statistically well‐balanced demographics observed between the groups (Figure 1A). The survival curves after matching, depicted in Figures 1B, reveal that patients in the uICI group had significantly better PFS compared to those in the uN group (2‐year PFS: 62.3% vs. 17.4%; *p* = 0.036), while no differences were observed between the uTKI and uN groups (2‐year PFS: 18.2% vs. 12.3%; *p* = 0.545). Regarding surgery‐related factors, there were no significant differences between the groups in blood loss volume and postoperative complication grades (Figure 1C). The postoperative pathological stages were compared between each group, but no significant differences were observed (Figure 1D). These results suggest that neoadjuvant systemic therapy does not contribute to the suppression of perioperative complications or downstaging of pathology; however, uICI combination therapy, rather than VEGFR‐targeted therapy, significantly improved PFS rates, indicating its potential efficacy.

When comparing the details of the treatment course between the uTKI and uICI groups (*n* = 23), as shown in Figure 1E, all uTKI patients experienced disease progression, unlike the uICI groups (chi‐square *p* = 0.005). Figure 1F depicts the maximum tumour reduction rate during systemic therapy and the reduction rate immediately before surgery, revealing that while there was no difference between the groups in the maximum reduction rate, the uICI group tended to maintain the reduction rate relatively well before surgery, whereas the uTKI group showed a tendency for stable or even enlargement of the tumour.

Our study has the following limitations: It was a retrospective observational study involving a small sample size, resulting in an inherent risk of selection and sampling bias, as the choice of systemic therapy or nephrectomy was determined by multiple physicians. To minimize potential bias, therefore, we employed propensity score matching to adjust for patient characteristics.

In conclusion, this study suggests that although there was no benefit for surgical and pathological outcomes, uICI combination therapy had significantly improved PFS rate as compared to upfront nephrectomy in unresectable/metastatic RCC patients, contrary to uTKI. Further studies with larger cohorts are needed to validate our results.

## AUTHOR CONTRIBUTIONS

**Sho Kiyota:** Investigation; methodology; formal analysis; data curation; visualization; writing—original draft. **Takashi Yoshida:** Conceptualization; project administration; investigation; methodology; writing—original draft; supervision. **Takahiro Nakamoto:** Data collection; writing—review and editing. **Eri Jino:** Data collection; review and editing. **Takao Mishima:** Review and editing; supervision. **Hidefumi Kinoshita:** Review and editing; supervision.

## CONFLICT OF INTEREST STATEMENT

None.

## ETHICS STATEMENT

The protocol for this research project has been approved by the Ethics Committee of Kansai Medical University, and it conforms to the provisions of the Declaration of Helsinki (ethics approval no. 2018109). In an opt‐out procedure, an individual is presumed to consent if they do not actively refuse the reuse of health data being applied.
